# Electronic Interactive Games for Glycemic Control in Individuals With Diabetes: Systematic Review and Meta-Analysis

**DOI:** 10.2196/43574

**Published:** 2024-02-12

**Authors:** WenQi Yao, YiBing Han, Li Yang, Ying Chen, ShengZhe Yan, YanZhen Cheng

**Affiliations:** 1 Department of Endocrinology Zhujiang Hospital of Southern Medical University Guangzhou China; 2 Department of Nutrition Zhujiang Hospital of Southern Medical University Guangzhou China

**Keywords:** electronic game, physical activity, diet, diabetes mellitus, glycemic control

## Abstract

**Background:**

Several electronic interventions have been used to improve glycemic control in patients with diabetes. Electronic interactive games specific to physical activity are available, but it is unclear if these are effective at improving glycemic control in patients with diabetes.

**Objective:**

This study aimed to determine the effects of electronic game–based interventions on glycemic control in patients with diabetes.

**Methods:**

Relevant studies that were published before April 1, 2023, were searched from 5 databases: PubMed, Embase, Web of Science, Scopus, and Cochrane Library. Eligibility criteria included prospective studies examining the relationship between electronic games with physical activities or diet education and glycemic control as the outcome. The risk of bias was assessed using the Cochrane risk-of-bias tool. All analyses were conducted using RevMan5.4.1. Depending on the heterogeneity across studies, the pooled effects were calculated using fixed-effects or random-effects models.

**Results:**

Participants from 9 studies were included and assessed. Glycated hemoglobin (HbA_1c_) and fasting blood glucose improved in the intervention group, although the analysis revealed no significant reduction in HbA_1c_ (−0.09%, 95% CI −0.29% to 0.10%) or fasting blood glucose (−0.94 mg/dL, 95% CI −9.34 to 7.46 mg/dL). However, the physical activity of individuals in the intervention group was significantly higher than that of those in the control group (standardized mean difference=0.84, 95% CI 0.30 to 1.38; *P*=.002). Other outcomes, such as weight and blood lipids, exhibited no significant improvement (all *P*>.05).

**Conclusions:**

Electronic games had a good impact on participants’ physical activity and offered an advantage in glycemic control without reaching statistical significance. Electronic games are convenient for reminders and education. Low-intensity exercise games may not be considered a better adjuvant intervention to improve diabetes self-management care.

## Introduction

Diabetes mellitus is one of the 4 major noncommunicable diseases and is also among the top 10 global causes of death. Throughout the world, the number of patients with diabetes mellitus is increasing, probably due to changes in lifestyle. According to the International Diabetes Federation, in 2021, approximately 536.6 million adults (aged 20-79 years) were living with diabetes; this is expected to rise to 12.2% in 2045 [1]. Because of the rise in type 1 and type 2 diabetes, the burden of health care expenditures and its complications continues to increase, whereas the complications are the main causes of morbidity and mortality [2]. To address the health challenge resulting from diabetes, effective and efficient management is needed [3-5].

Lifestyle management, an efficacious method for diabetes prevention [6], is a fundamental aspect of diabetes care. It includes diabetes self-management education and support, medical nutrition therapy, physical activity, smoking cessation counseling, and psychosocial care [7]. Food intake and physical activity are associated with significantly improved control of diabetes [8]. With advances in technology, lifestyle management incorporating novel technologies and formats meets the needs of various populations for diabetes treatment [9]. New methods, such as electronic games and wearable devices, aim to contribute to better patient compliance [10].

It has been reported that electronic games can help players learn more about healthy diets and encourage exercise [11,12]. Although they play a role as facilitators in motivating and accelerating physical activity, they offer little benefit to patients with chronic disease [13]. Previous systematic reviews have evaluated the impact of app-based or electronic health interventions to support changes in blood glucose management, physical activity, or diet [9,14,15]. However, previous papers analyzed relatively few articles or articles that were not solely on using games. They also used educational or regulation applications, robots, or virtual worlds that do not contain game elements. Electronic games specific to physical activity and dietary education are available; however, we currently lack an understanding of how effective electronic games can be for glycemic control.

In this study, we performed a comprehensive literature search to select studies on the effects of electronic game–based interventions on glycemic control in patients with diabetes for meta-analysis. Electronic gaming interventions are defined as containing an element of gaming that involves virtual reality, serious gaming, or exergaming [15].

## Methods

### Data Sources and Search Strategy

This review was conducted in accordance with the PRISMA (Preferred Reporting Items for Systematic Reviews and Meta-Analyses) statement and its associated checklist (Multimedia Appendix 1). Relevant studies that were published before April 1, 2023, were searched from 5 databases: PubMed, Embase, Web of Science, Scopus, and Cochrane Library. The references of the included studies were hand-searched to identify any additional articles. The following terms were used during the search: (“Diabetes” OR “diabetic” OR “diabetes mellitus” OR “glycemic control” OR “glucose control” OR “glucose”) AND (“game” OR “gamification” OR “exergaming” OR “avatar” OR “wii” OR “virtual” OR “konami” OR “wii-fit” OR “kinect” OR “tierone” OR “video-game” OR “serious-games” OR “serious video-games” OR “Augmented reality” OR “mixed reality” OR “second life” OR “TierOne” OR “Konami Dance Dance Revolution” OR “Sony Eyetoy” OR “Microsoft Kinec”). Detailed search strategies for each database are given in Multimedia Appendix 2. The reference lists of the searched articles and the relevant reviews were then screened to identify any pertinent studies.

### Study Selection

Studies included in this meta-analysis met the following criteria: (1) participants were diagnosed as having type 1 diabetes or type 2 diabetes; (2) the articles were published in English or Chinese; (3) the articles presented the electronic management intervention with a gaming element, such as a virtual reality game, serious game, or exergame; and (4) the outcome indicators were blood glucose and glycated hemoglobin (HbA_1c_).

Studies that met the following criteria were excluded: (1) participants had gestational diabetes mellitus, had other special types of diabetes mellitus, underwent surgery, had an operation, or were in the emergency department; (2) participants had a previous history of mental illnesses, eating disorders, or cancer; (3) the management intervention was only based on an online, mobile, or virtual application but did not use a gaming element; and (4) articles that were protocols, conference abstracts, case reports, reviews, or meta-analyses.

Articles were screened in a 2-step process. First, all titles and abstracts were examined by 2 investigators. Any citations that clearly did not meet the inclusion criteria were excluded. Second, all abstracts and full-text articles were examined independently by 2 investigators. Any disagreements in the selection process were resolved through discussion with a third investigator.

### Risk of Bias

The included trials were independently assessed by 2 investigators for the risk of bias using the Cochrane risk-of-bias tool [16]. An assessment was performed across 5 domains of bias (sequence generation, allocation concealment, blinding, incomplete outcome data, and selective reporting). The risk of bias was assessed as either low (proper methods taken to reduce bias), high (improper methods creating bias), or unclear (insufficient information provided to determine the bias level). All discrepancies and disagreements were resolved through consensus or, where necessary, by a third author.

### Data Extraction

A Microsoft Excel table was used to extract data on the year of publication, country, sample size, participant characteristics, study setting and design, intervention and control arms, duration, and outcome data. The main outcomes included HbA_1c_ or fasting blood glucose (FBG). The secondary outcomes included daily steps (regarded as a physical activity outcome), blood pressure, and weight, among others. The data were obtained from the original text and attachments supplied. Data from different studies were converted to common units. Data extraction was carried out by 2 reviewers independently. All discrepancies and disagreements were resolved through consensus.

### Missing Data

Study authors were contacted by email where there were missing or unclear data (for instance, relating to the primary outcome). Studies for which insufficient primary data were available (eg, missing data cannot be obtained) were excluded from analysis but not from the review.

### Data Synthesis and Quality Assessment

All analyses were conducted using RevMan5.4.1 (Cochrane). Data were expressed as the mean difference (MD) and 95% CI and pooled using fixed-effect or random-effects models according to the heterogeneity. A random-effects model assumes that the study estimates are estimating different, yet related, intervention effects and thus incorporates the heterogeneity among studies. This is a more appropriate method to pool studies that may differ slightly in the distribution of risk factors, population, size, and outcomes.

Heterogeneity was assessed using a χ^2^ test and quantified using the *I*^2^ statistic. Significance for the heterogeneity was set at *P*<.05, with an *I*^2^>50% considered to be evidence of high heterogeneity, which prompted us to use the random-effects model to pool the data.

## Results

### Overview

**Figure 1:**
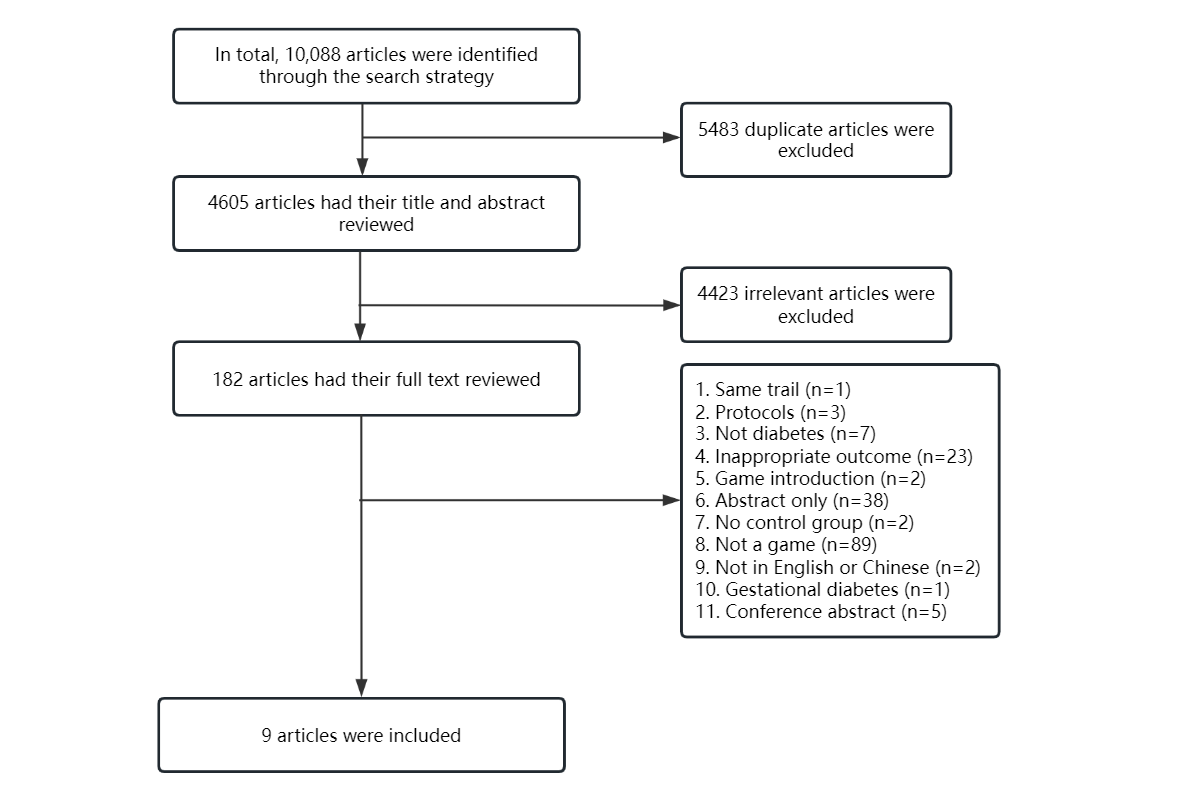
Flowchart of the study selection process.

Our search identified 10,088 articles, of which 4605 were screened after removing duplicate records. Of these, 182 were identified for further evaluation. Of these, 173 were excluded, resulting in 9 included studies (Figure 1). Of the excluded articles, 18 were excluded because they only had abstracts and we could not access the original text and data.

The results of the remaining 9 studies, comprising 913 participants and 747 cases of type 2 diabetes, were included in the meta-analysis [17-25]. The characteristics of all 9 studies are shown in Multimedia Appendix 3 [17-25]. The duration of trials ranged from 1 month to 1 year. Of the 9 studies, 4 were undertaken in the United States [18,21,23,25], 2 in Europe [17,20], and 3 in Asia [19,22,24]. Of the 9 studies, 3 assessed FBG and 8 assessed HbA_1c_.

**Figure 2:**
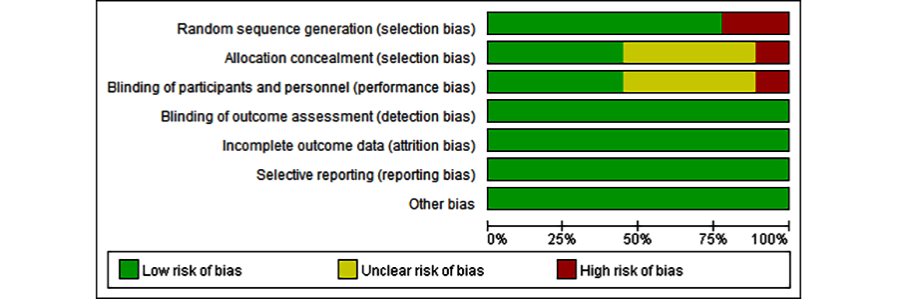
Risk-of-bias graph showing the authors’ judgments about each risk-of-bias item presented as percentages across all the included studies. A total of 9 trials were assessed for risk of bias.

**Figure 3:**
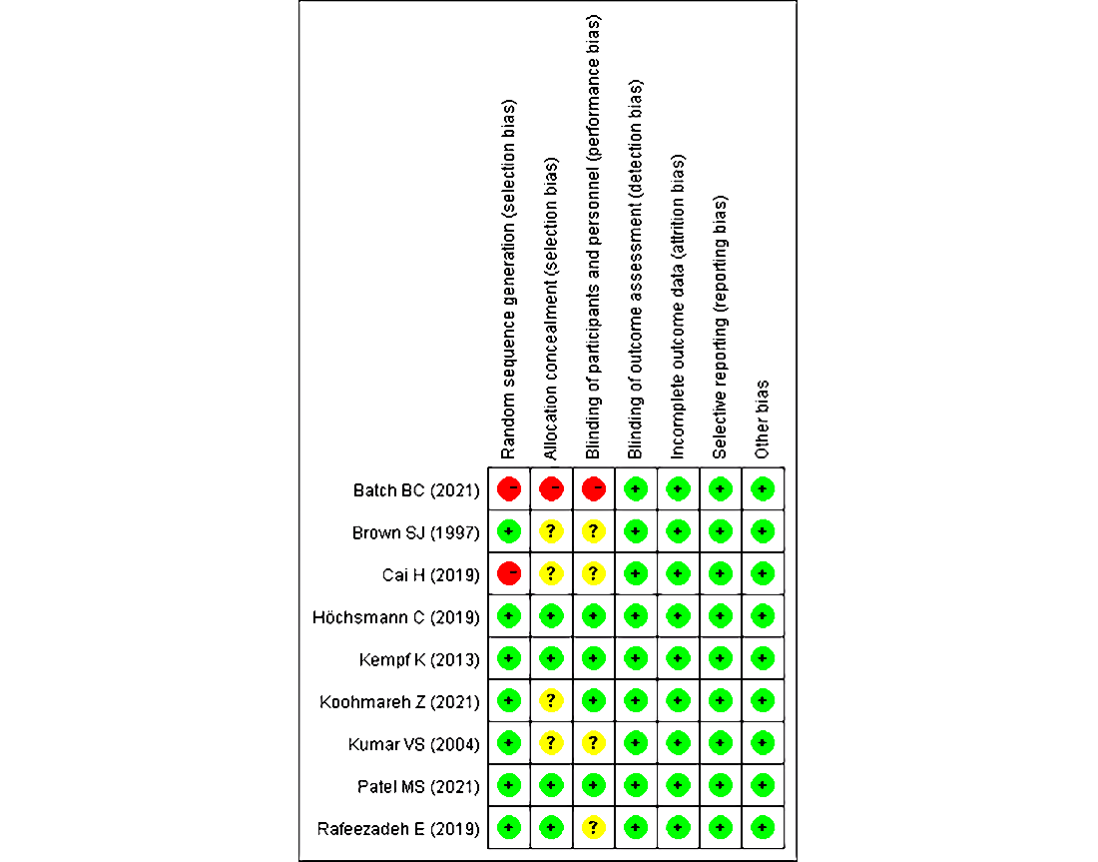
Risk-of-bias summary showing the authors’ judgments about each risk-of-bias item for each included study. Green "+": low risk of bias; red "–": high risk of bias; yellow "?": unknown risk of bias.

The studies included 2 non–randomized controlled trials (RCTs) and 7 RCTs, the quality of which was assessed using the Cochrane risk-of-bias tool. We determined that 3 studies were of high quality, whereas 4 were of moderate quality and 2 were of low quality (Figures 2 and 3 [17-25]). The 2 non-RCTs were not random and the allocations were unclear. Blinding was difficult in game interventions; 1 study was unblinded [25] and 4 were unclear, but the studies made an effort to blind either patients or personnel. One study was not blinded to the outcome assessment, but it was still analyzed as low risk, considering its main outcome was the objective index. The 9 studies had no elective outcome reporting.

Publication bias was not assessed for any outcome as <10 trials were available.

### Meta-Analysis

#### HbA_1c_ Level

A total of 8 articles had HbA_1c_ testing but 1 did not provide postintervention data [25]. We sent an email to the author with a request to provide the raw data but received no reply.

**Figure 4:**
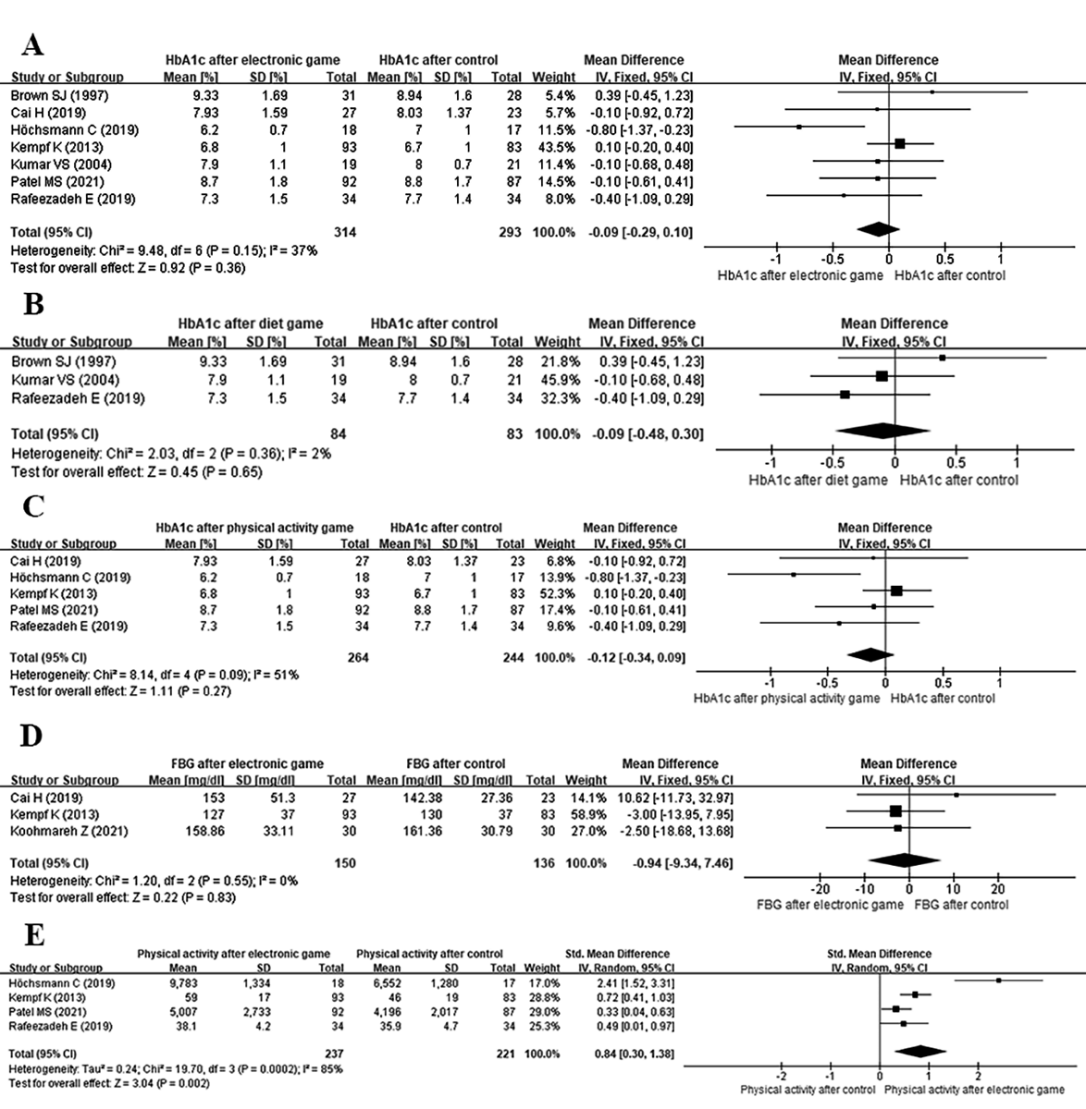
Meta-analysis of the effect of electronic games on HbA_1c_, FBG, and physical activity. (A) HbA_1c_ after a diet intervention or physical activity intervention; (B) HbA_1c_ after a diet intervention; (C) HbA_1c_ after a physical activity game intervention; (D) FBG after an electronic game intervention; and (E) physical activity after an electronic game intervention. FBG: fasting blood glucose; HbA_1c_: glycated hemoglobin; IV: inverse variance; Std.: standardized.

As shown in Figure 4A [17,21,23-25], this analysis showed a clinically important improvement in HbA_1c_, but there was no significant reduction after the intervention among individuals with diabetes mellitus (7 studies; n=607; MD=−0.09%, 95% CI −0.29% to 0.10%; *I*^2^=37%; *P*=.36). Figure 4B shows the change in HbA_1c_ after a diet-based game intervention (3 studies; n=167; MD=−0.09%, 95% CI −0.48% to 0.30%; *I*^2^=2%; *P*=.65). Figure 4C shows the change in HbA_1c_ after a physical activity–based game intervention (5 studies; n=508, MD=−0.12%, 95% CI −0.34% to 0.09%; *I*^2^=51%; *P*=.27).

#### Fasting Blood Glucose Level

The meta-analysis showed that the FBG level of the intervention groups was not statistically different from that of the control groups (3 studies; n=286; MD=−0.94 mg/dL, 95% CI −9.34 to 7.46 mg/dL; *I*^2^=0%, *P*=.83; Figure 4D).

#### Physical Activity

Of the 7 RCTs, 2 assessed self-reported physical activity and 2 counted participants’ daily steps during the intervention to assess the patients’ physical activity. Because of the differences in measurement instruments, we calculated standardized mean differences (SMDs). These results were statistically heterogeneous with respect to the effect (χ^2^_3_=19.70; *P*<.001; *I*^2^=85%); we found a significant increase in physical activity above baseline in the intervention groups. Moreover, participants assigned to the intervention groups increased their physical activity significantly more than participants in the control groups (SMD=0.84; 95% CI 0.30 to 1.38; *P*=.002; Figure 4E).

#### Weight

**Figure 5:**
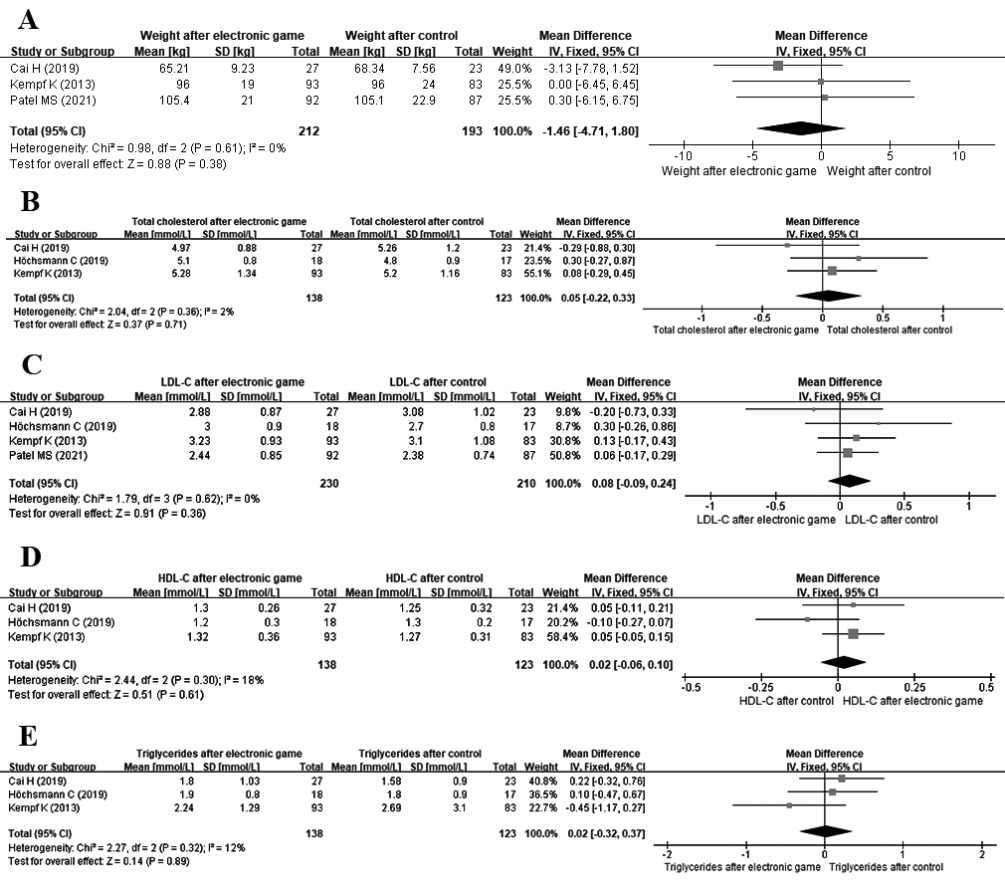
Meta-analysis of the effect of electronic games on (A) weight, (B) total cholesterol, (C) LDL-C, (D) HDL-C, and (E) triglycerides. HDL-C: high-density lipoprotein cholesterol; IV: inverse variance; LDL-C: low-density lipoprotein cholesterol; Std.: standardized.

Weight also trended toward decreases in the intervention groups, with an MD of −1.46 kg (95% CI −4.71 to 1.80 kg; Figure 5A [17-19]). However, the decreases did not reach statistical significance (*P*=.38).

#### Blood Lipids

There was no significant reduction in total cholesterol (3 studies; n=261; MD=0.05 mmol/L, 95% CI −0.22 to 0.33 mmol/L; *I*^2^=2%; *P*=.71; Figure 5B), low-density lipoprotein cholesterol (4 studies; n=440; MD=0.08 mmol/L, 95% CI −0.09 to 0.24 mmol/L; *I*^2^=0%; *P*=.36; Figure 5C), high-density lipoprotein cholesterol (3 studies; n=261; MD=0.02 mmol/L, 95% CI −0.06 to 0.10 mmol/L; *I*^2^=18%; *P*=.61; Figure 5D), or triglycerides (3 studies; n=261; MD=0.02 mmol/L, 95% CI −0.32 to 0.37 mmol/L; *I*^2^=12%; *P*=.89; Figure 5E) after the intervention among patients with diabetes mellitus.

## Discussion

### Principal Findings

This study demonstrated that electronic interactive games were associated with a good impact on participants’ physical activity. However, we found that electronic interactive games did not present a significant benefit for HbA_1c_ levels, FBG levels, weight, or blood lipids compared to the control group. The game interventions were intended for education to manage diabetes through games.

### Effects of Diet Education Games on Blood Glucose

Plant-based diets and exercise are major diabetes-protective factors [26]. The Da Qing Diabetes Prevention Study showed an overall 51% reduction in diabetes incidence in participants after a 6-year intervention with diet, exercise, or both; its 30-year follow-up showed that lifestyle interventions reduced the incidence of serious diabetes complications and diabetes-related mortality [27]. However, Hemmingsen et al [28] did not find firm evidence that diet alone or physical activity alone influences the risk of type 2 diabetes mellitus or its associated complications in people at increased risk of developing type 2 diabetes mellitus compared to standard treatment [28]. The trials included in this study had little data on the impact of games on diet, and only 3 articles evaluated participants’ postintervention diet. From the results, education through games was effective, although the improvements in glycemic control were not statistically significant. The most important reason was that the 3 trials studied patients with type 1 diabetes mellitus aged 8 to 18 years. The games provided diabetes-related diet education to the patients, but family-based diet intervention may also not impact glycemic control [29].

### Effects of Games Related to Physical Activity on Blood Glucose

Physical activity with different intensities impacts glycemic control in individuals with diabetes. Of the included studies, 4 trials [17,18,20,24] assessed physical activity by daily steps or self-reported activity, and this analysis found a significant increase. These results are consistent with findings from other meta-analyses showing increased physical activity among patients with chronic disease [30-32]. Some studies find positive effects with low-intensity physical activity, although these are not reflected by a decrease in HbA_1c_ or FBG in patients with type 2 diabetes [33-35]. A meta-analysis showed that high-intensity interval exercise significantly reduced HbA_1c_ levels compared to no or low-intensity exercise [36]. Low exercise intensity in the 9 studies we included may be the reason why there was no significant difference in HbA_1c_ and FBG in patients with diabetes between the groups. However, the games in the virtual reality group were relatively novel, which was very helpful for improving cognition, physical skills that are directly involved in functional abilities, and enthusiasm for sports [19].

The study by Höchsmann et al [20] contributed a substantial amount of heterogeneity; without this study, *I*^2^ was 11%. The high heterogeneity may have been caused by the baseline of the participants in this trial being better than those in the other trials. In their trial, Höchsmann et al [20] used a dilapidated garden to symbolize the patient’s physical condition, and exercise and daily physical activity execution were tracked by mobile phones, allowing for feedback. After 24 weeks of intervention, there was no significant change in HbA_1c_ levels in the intervention group, while HbA_1c_ levels in the control group receiving 1-time lifestyle counseling increased. In the trial, the intervention group had a higher increase in daily steps than the control group, providing evidence that physical activity can be encouraged by electronic games.

### Effects of Games on Blood Lipids, Blood Pressure, and BMI

In our study, game-based intervention resulted in no significant decrease in blood lipids in patients with diabetes. Only 2 trials reported the outcomes of blood pressure [17,20] and BMI [17,19], and the 2 indexes were both reduced. Systolic blood pressure was below 140 mm Hg but above 130 mm Hg, which is still high for patients with diabetes. Treatment with medication may be indispensable.

### Effects of Games on Weight

Lifestyle intervention can be effective for achieving clinically important reductions in body weight [37,38]. It has been demonstrated that electronic game activities are engaging, which encourages their use on a regular basis, improving the long-term outcome of a treatment for obesity [39,40]. However, an intervention using a different avatar did not improve physical activity practice or self-efficacy expectations [41]. Gomez et al [42] showed that high exercise intensity from active electronic games elicited significant increases in energy expenditure. In this study, electronic games did not result in significant weight reduction, and BMI was reduced slightly in 2 trials. Possible reasons include insufficient physical activity and that participants did not strictly control their diet. Whether electronic games are beneficial for weight control by encouraging appropriate intensity exercise in patients with diabetes requires more clinical evidence in the future.

The reasons for the lack of significant results in this meta-analysis may be as follows. First, participants in the control groups were also familiar with what the game taught. Second, patients with type 2 diabetes mellitus in the intervention groups, who were all older than 40 years, could not make full use of electronic devices and adapt to the games. Third, for exercise-based interventions, not all studies involved regular exercise monitoring for participants and established appropriate feedback or interaction mechanisms.

### Limitations and Future Directions

This study had several limitations. First, not every included study reported the HbA_1c_ and FBG levels. Some excluded studies had relevant interventions but did not observe blood glucose changes or failed to give detailed trial data results. Second, the studies that were included in this meta-analysis were not homogeneous. Different games or game mechanisms were used in different patient populations. The number of participants was not large in several of the included studies, and each study used different games. Therefore, it is difficult to conduct detailed hierarchical verification of the effects of different games on blood glucose. We strived to ensure that the included studies were high-quality RCTs with strict inclusion and exclusion criteria, excluding nongaming electronic interventions. Existing studies have evaluated the effectiveness of electronic games as an alternative for traditional diabetes education. As diabetes continues, it is necessary to promote this management model. However, future studies should not only design the game in terms of increased knowledge and improved self-management but should encourage enhanced physical activity intensity.

### Conclusion

As an alternative treatment tool in diabetes management, the studies on electronic games explored in this study showed a clinical improvement in glycemic control and weight control, although this improvement was not superior to that observed in the control participants. Thus, such interventions may complement existing treatment courses for diet, self-management education, and high-intensity physical activity to potentially increase the compliance of patients with diabetes. More new technologies can be used for diabetes control, and electronic games can be designed for different groups of patients with diabetes. For example, immersive virtual reality is an emerging strategy to enhance exercise performance for young patients with diabetes, and the metaverse may be a new community enabling older patients to form new social connections and share their experiences of living with diabetes. Interactive exercise games can be used in children to increase interest in education and family companionship time, and thus improve exercise compliance.

## Appendix

Multimedia Appendix 1 PRISMA (Preferred Reporting Items for Systematic Reviews and Meta-Analyses) 2020 checklist.

Multimedia Appendix 2 Search strategy.

Multimedia Appendix 3 Basic characteristics of the included studies.

## Abbreviations

FBG: fasting blood glucose
HbA_1c_: glycated hemoglobin
MD: mean difference
PRISMA: Preferred Reporting Items for Systematic Reviews and Meta-Analyses
RCT: randomized controlled trial
SMD: standardized mean difference

## References

[1] Diabetes around the world in 2021. IDF Diabetes Atlas.

[2] Diabetes. World Health Organization.

[3] Gregg EW, Sattar N, Ali MK (2016). The changing face of diabetes complications. Lancet Diabetes Endocrinol.

[4] Molania T, Alimohammadi M, Akha O, Mousavi J, Razvini R, Salehi M (2017). The effect of xerostomia and hyposalivation on the quality of life of patients with type II diabetes mellitus. Electron Physician.

[5] Zheng Y, Ley SH, Hu FB (2018). Global aetiology and epidemiology of type 2 diabetes mellitus and its complications. Nat Rev Endocrinol.

[6] Pan X, Li G, Hu Y, Wang J, Yang W, An Z, Hu Z, Lin J, Xiao J, Cao H, Liu P, Jiang X, Jiang Y, Wang J, Zheng H, Zhang H, Bennett PH, Howard BV (1997). Effects of diet and exercise in preventing NIDDM in people with impaired glucose tolerance. the Da Qing IGT and Diabetes Study. Diabetes Care.

[7] American Diabetes Association (2019). 5. Lifestyle management: standards of medical care in diabetes-2019. Diabetes Care.

[8] Liu M, Liu C, Zhang Z, Zhou C, Li Q, He P, Zhang Y, Li H, Qin X (2021). Quantity and variety of food groups consumption and the risk of diabetes in adults: a prospective cohort study. Clin Nutr.

[9] Weber MB, Hassan S, Quarells R, Shah M (2021). Prevention of type 2 diabetes. Endocrinol Metab Clin North Am.

[10] Cahn A, Akirov A, Raz I (2018). Digital health technology and diabetes management. J Diabetes.

[11] Kerfoot BP, Gagnon DR, McMahon GT, Orlander JD, Kurgansky KE, Conlin PR (2017). A team-based online game improves blood glucose control in veterans with type 2 diabetes: a randomized controlled trial. Diabetes Care.

[12] Ruppert B (2011). New directions in virtual environments and gaming to address obesity and diabetes: industry perspective. J Diabetes Sci Technol.

[13] Jo A, Coronel BD, Coakes CE, Mainous AG (2019). Is there a benefit to patients using wearable devices such as Fitbit or health apps on mobiles? a systematic review. Am J Med.

[14] Martos-Cabrera MB, Membrive-Jiménez MJ, Suleiman-Martos N, Mota-Romero E, Cañadas-de la Fuente GA, Gómez-Urquiza JL, Albendín-García L (2020). Games and health education for diabetes control: a systematic review with meta-analysis. Healthcare (Basel).

[15] Christensen J, Valentiner LS, Petersen RJ, Langberg H (2016). The effect of game-based interventions in rehabilitation of diabetics: a systematic review and meta-analysis. Telemed e-Health.

[16] Higgins JPT, Altman DG, Gøtzsche PC, Jüni P, Moher D, Oxman AD, Savovic J, Schulz KF, Weeks L, Sterne JAC (2011). The Cochrane Collaboration's tool for assessing risk of bias in randomised trials. BMJ.

[17] Kempf K, Martin S (2013). Autonomous exercise game use improves metabolic control and quality of life in type 2 diabetes patients - a randomized controlled trial. BMC Endocr Disord.

[18] Patel MS, Small DS, Harrison JD, Hilbert V, Fortunato MP, Oon AL, Rareshide CAL, Volpp KG (2021). Effect of behaviorally designed gamification with social incentives on lifestyle modification among adults with uncontrolled diabetes: a randomized clinical trial. JAMA Netw Open.

[19] Cai H, Li G, Jiang S, Yin H, Liu P, Chen L (2019). Effect of low-intensity, Kinect-based Kaimai-style qigong exercise in older adults with type 2 diabetes. J Gerontol Nurs.

[20] Höchsmann C, Müller O, Ambühl M, Klenk C, Königstein K, Infanger D, Walz SP, Schmidt-Trucksäss A (2019). Novel smartphone game improves physical activity behavior in type 2 diabetes. Am J Prev Med.

[21] Brown SJ, Lieberman DA, Germeny BA, Fan YC, Wilson DM, Pasta DJ (1997). Educational video game for juvenile diabetes: results of a controlled trial. Med Inform (Lond).

[22] Koohmareh Z, Karandish M, Hadianfard AM (2021). Effect of implementing a mobile game on improving dietary information in diabetic patients. Med J Islam Repub Iran.

[23] Kumar VS, Wentzell KJ, Mikkelsen T, Pentland A, Laffel LM (2004). The DAILY (Daily Automated Intensive Log for Youth) trial: a wireless, portable system to improve adherence and glycemic control in youth with diabetes. Diabetes Technol Ther.

[24] Rafeezadeh E, Ghaemi N, Miri HH, Rezaeian A (2019). Effect of an educational video game for diabetes self-management on adherence to a self-care regimen in children with type 1 diabetes. Evid Based Care J.

[25] Batch BC, Spratt SE, Blalock DV, Benditz C, Weiss A, Dolor RJ, Cho AH (2021). General behavioral engagement and changes in clinical and cognitive outcomes of patients with type 2 diabetes using the Time2Focus mobile app for diabetes education: pilot evaluation. J Med Internet Res.

[26] Kolb H, Martin S (2017). Environmental/lifestyle factors in the pathogenesis and prevention of type 2 diabetes. BMC Med.

[27] Gong Q, Zhang P, Wang J, Ma J, An Y, Chen Y, Zhang B, Feng X, Li H, Chen X, Cheng Y J, Gregg E W, Hu Y, Bennett PH, Li G (2019). Morbidity and mortality after lifestyle intervention for people with impaired glucose tolerance: 30-year results of the Da Qing Diabetes Prevention Outcome Study. The Lancet Diabetes & Endocrinology.

[28] Hemmingsen B, Gimenez-Perez G, Mauricio D, Roqué IFM, Metzendorf MI, Richter B (2017). Diet, physical activity or both for prevention or delay of type 2 diabetes mellitus and its associated complications in people at increased risk of developing type 2 diabetes mellitus. Cochrane Database Syst Rev.

[29] Nansel TR, Laffel LMB, Haynie DL, Mehta SN, Lipsky LM, Volkening LK, Butler DA, Higgins LA, Liu A (2015). Improving dietary quality in youth with type 1 diabetes: randomized clinical trial of a family-based behavioral intervention. Int J Behav Nutr Phys Act.

[30] Hodkinson A, Kontopantelis E, Adeniji C, van Marwijk H, McMillian B, Bower P, Panagioti M (2021). Interventions using wearable physical activity trackers among adults with cardiometabolic conditions: a systematic review and meta-analysis. JAMA Netw Open.

[31] Qiu S, Cai X, Chen X, Yang B, Sun Z (2014). Step counter use in type 2 diabetes: a meta-analysis of randomized controlled trials. BMC Med.

[32] Martos-Cabrera MB, Velando-Soriano A, Pradas-Hernández L, Suleiman-Martos N, Cañadas-de la Fuente GA, Albendín-García L, Gómez-Urquiza JL (2020). Smartphones and apps to control glycosylated hemoglobin (HbA1c) level in diabetes: a systematic review and meta-analysis. J Clin Med.

[33] Reiner M, Niermann C, Jekauc D, Woll A (2013). Long-term health benefits of physical activity--a systematic review of longitudinal studies. BMC Public Health.

[34] Piercy KL, Troiano RP, Ballard RM, Carlson SA, Fulton JE, Galuska DA, George SM, Olson RD (2018). The physical activity guidelines for Americans. JAMA.

[35] Lee I, Shiroma EJ, Kamada M, Bassett DR, Matthews CE, Buring JE (2019). Association of step volume and intensity with all-cause mortality in older women. JAMA Intern Med.

[36] Ruffino JS, Songsorn P, Haggett M, Edmonds D, Robinson AM, Thompson D, Vollaard NB (2017). A comparison of the health benefits of reduced-exertion high-intensity interval training (REHIT) and moderate-intensity walking in type 2 diabetes patients. Appl Physiol Nutr Metab.

[37] Franz MJ, Boucher JL, Rutten-Ramos S, VanWormer JJ (2015). Lifestyle weight-loss intervention outcomes in overweight and obese adults with type 2 diabetes: a systematic review and meta-analysis of randomized clinical trials. J Acad Nutr Diet.

[38] Salinardi TC, Batra P, Roberts SB, Urban LE, Robinson LM, Pittas AG, Lichtenstein AH, Deckersbach T, Saltzman E, Das SK (2013). Lifestyle intervention reduces body weight and improves cardiometabolic risk factors in worksites. Am J Clin Nutr.

[39] Skip Rizzo A, Lange B, Suma EA, Bolas M (2011). Virtual reality and interactive digital game technology: new tools to address obesity and diabetes. J Diabetes Sci Technol.

[40] Cesa GL, Manzoni GM, Bacchetta M, Castelnuovo G, Conti S, Gaggioli A, Mantovani F, Molinari E, Cárdenas-López G, Riva G (2013). Virtual reality for enhancing the cognitive behavioral treatment of obesity with binge eating disorder: randomized controlled study with one-year follow-up. J Med Internet Res.

[41] Navarro J, Cebolla A, Llorens R, Borrego A, Baños RM (2020). Manipulating self-avatar body dimensions in virtual worlds to complement an internet-delivered intervention to increase physical activity in overweight women. Int J Environ Res Public Health.

[42] Gomez DH, Bagley JR, Bolter N, Kern M, Lee CM (2018). Metabolic cost and exercise intensity during active virtual reality gaming. Games Health J.

