# Supplementary material for: Electronic Interactive Games for Glycemic Control in Individuals With Diabetes: Systematic Review and Meta-Analysis
Source: JMIR Serious Games. 2024 Feb 12;12:e43574. doi: 10.2196/43574 (PMC10897792; doi:10.2196/43574)
Supplement: Multimedia Appendix 2 [file games_v12i1e43574_app2.pdf]

e-Methods: Search strategy

PubMed:

1. "Diabetes Mellitus"[Mesh] OR ("Diabetes" OR "diabetic" OR "diabetes mellitus"
OR
"glycemic control" OR "glucose control" OR "glucose")[Title/Abstract]
2. "Games, Experimental"[Mesh] OR "Gamification"[Mesh] OR "Video Games"[Mesh] OR
"Virtual
Reality
Exposure
Therapy"[Mesh]
OR
"Virtual
Reality"[Mesh]
OR
"Exergaming"[Mesh] OR "Telerehabilitation"[Mesh] OR "Augmented Reality"[Mesh]
3. ("game" OR "gamification" OR "exergaming" OR "avatar" OR "virtual" OR "video-game" OR "serious video-games" OR "Augmented reality" OR "mixed reality")[Title/Abstract]
4. "wii" OR "konami" OR "wii-fit" OR "kinect" OR "tierone" OR "second life" OR "TierOne" OR "Konami Dance Dance Revolution" OR "Sony Eyetoy" OR "Microsoft Kinec"
5. 2 or 3 or 4
6. 1 and 5
7.
("Surgical Procedures, Operative"[Mesh] OR Operat*[Title/Abstract] OR Surgery [Title/Abstract] OR Surgical [Title/Abstract])
8. 6 not 7, Filters: Humans, Chinese, English

Embase:

1. ("Diabetes Mellitus" OR "Diabetes" OR "diabetic" OR "diabetes mellitus" OR "glycemic control" OR "glucose control" OR "glucose"):ab,ti
2.
(("Games, Experimental" OR "Gamification" OR "Video Games" OR "Virtual Reality Exposure Therapy" OR "Virtual Reality" OR "Exergaming" OR "Telerehabilitation" OR "Augmented Reality" OR "game" OR "gamification" OR "avatar" OR "virtual" OR "mixed reality" OR "wii" OR "konami" OR "wii-fit" OR "kinect" OR "tierone" OR "second life" OR "TierOne" OR "Konami Dance Dance Revolution" OR "Sony Eyetoy" OR "Microsoft Kinec"):ab,ti) NOT ((Operat* OR Surgery OR Surgical):ab,ti)
3. 1 and 2

Wed of Science:

1. topic: ("Diabetes Mellitus" or"Diabetes" OR "diabetic" OR "diabetes mellitus" OR "glycemic control" OR "glucose control" OR "glucose")
2. topic: ("Games, Experimental" OR "Gamification"OR "Video Games" OR "Virtual Reality Exposure Therapy" OR "Virtual Reality"OR "Exergaming" OR "Telerehabilitation" OR "Augmented Reality" OR "game" OR "gamification" OR "exergaming" OR "avatar" OR "virtual" OR "video-game" OR "serious video-games"OR "Augmented reality" OR "mixed reality" OR"wii" OR "konami" OR "wii-fit" OR "kinect" OR "tierone" OR "second

life" OR "TierOne" OR "Konami Dance Dance Revolution" OR "Sony Eyetoy" OR "Microsoft Kinec")

3. topic: (Operat* OR Surgery OR Surgical)

4. 1 and 2 not 3

Scopus:1. ("Diabetes Mellitus" OR "Diabetes" OR "diabetic" OR "diabetes mellitus" OR "glycemic control" OR "glucose control" OR "glucose") AND (LIMIT-TO (LANGUAGE, "English") OR LIMIT-TO (LANGUAGE , "Chinese"))

2. "Games, Experimental" OR "Gamification" OR "Video Games" OR "Exergaming" OR "Telerehabilitation" OR "game" OR "gamification" OR "avatar" OR "video-game" OR "serious video-games" OR "Virtual Reality Exposure Therapy" OR "Virtual Reality" OR "virtual" OR "Augmented reality" OR "mixed reality"OR"wii" OR "konami" OR "wii-fit" OR "kinect" OR "tierone" OR "second life" OR "TierOne" OR "Konami Dance Dance Revolution" OR "Sony Eyetoy" OR "Microsoft Kinec"

3. 1 AND 2 AND NOT (Operation OR Surgery OR Surgical)

Cochrane Library:

1. MeSH descriptor: [Diabetes Mellitus] explode all trees
2. ("Diabetes" OR "diabetic" OR "diabetes mellitus" OR "glycemic control" OR "glucose control" OR "glucose"):ti,ab,kw
3. 1 or 2
4. MeSH descriptor: [Gamification] explode all trees
5. MeSH descriptor: [Video Games] explode all trees
6. MeSH descriptor: [Games, Experimental] explode all trees
7. MeSH descriptor: [Virtual Reality Exposure Therapy] explode all trees
8. MeSH descriptor: [Virtual Reality] explode all trees
9. MeSH descriptor: [Augmented Reality] explode all trees
10. "game" OR "gamification" OR "exergaming" OR "avatar" OR "virtual" OR "video game" OR "Augmented reality" OR "mixed reality" OR "wii" OR "konami" OR "wii-fit" OR "kinect" OR "tierone" OR "second life" OR "TierOne" OR "Konami Dance Dance Revolution" OR "Sony Eyetoy" OR "Microsoft Kinec"
11. 4 OR 5 OR 6 OR 7 OR 8 OR 9 OR 10
12. MeSH descriptor: [Surgical Procedures, Operative] explode all trees
13. (Surgery OR Surgical):ti,ab,kw
14. 12 or 13
15. 3 and 11
16. 15 not 14
