# Supplementary material for: Electronic Interactive Games for Glycemic Control in Individuals With Diabetes: Systematic Review and Meta-Analysis
Source: JMIR Serious Games. 2024 Feb 12;12:e43574. doi: 10.2196/43574 (PMC10897792; doi:10.2196/43574)
Supplement: Multimedia Appendix 3 [file games_v12i1e43574_app3.docx]

**Multimedia Appendix 3.** Basic characteristics of the included studies.

| Author, Year (Country) | Study Design | Participants | Sample Size; Age | Intervention | Comparison | Duration and Frequency；Assessment Timepoints | Outcomes |
| --- | --- | --- | --- | --- | --- | --- | --- |
| Kempf K et al, 2013 (Germany) [17] | 2-arm  Randomized  Trial | T2DM subscribed in the disease management program (DMP);  BMI ≥27 kg/m^2^;  no regular physical activity;  no pharmacological diabetes therapy with the exception of Metformin or DPP-4 inhibitors. | Sample Size:  Pre-intervention: N = 220 (I: N = 120;  C: N = 100)  Post-intervention assessment:  N = 147 (I: N = 93; C: N =83);  Mean Age ± SD:  (N = 220) Total: 50-75 year-old  I: 62 ± 11 year-old;  C: 60 ± 9 year-old. | Use these tools(a Wii console, a balance board and the exercise game Wii Fit Plus) for at least 30 min/day; | Routine care (i.e. quarterly visits to the attending physician  according to the DMP T2DM with check of HbA1c,  weight, BMI, blood pressure, and blood lipids and therapy  adjustment if necessary) | Duration: 12 weeks  Assessment Timepoints :Baseline and after  the 12-weeks  intervention | Glycosylated hemoglobin (HbA1c); Fasting blood glucose (FBG); Weight; Body mass index(BMI); Total cholesterol(TC); Low-density lipoprotein cholesterol(LDL-C);  High-density lipoprotein cholesterol(HDL-C);  Triglycerides(TG); Self-reported  physical activity |
| Patel MS et al, 2021 (USA) [18] | 4-arm  Randomized  Trial | Type 2 diabetes;  the most recent HbA1c ≥8.0%;  BMI ≥ 25. | Sample Size:  Pre-intervention: I: N =87; C: N = 87;  Post-intervention assessment: I: N =80; C: N = 84;  Mean Age ± SD:  I: 51.3 ±10.6year-old;  C: 53.4 ±10.6 year-old. | Received education on the importance of diet and physical activity for weight loss and glycemic control.  Entered into a game with points and received an email with a leaderboard that ranked them on their cumulative points in the study thus far and displayed their level. | Received education on the importance of diet and physical activity for weight loss and glycemic control.  Received regular feedback and goal setting from the devices and smartphone application but received no other interventions. | Duration:  12 months  Assessment Timepoints :Baseline and after the 12 months intervention. | Weight;  HbA1c;  LDL-C level;  daily steps. |
| Cai H et al, 2019( China) [19] | 2-arm non-randomized controlled  trial | Diagnosed T2DM for⩽10 years;  without moderate or strenuous exercise 6 months;  without practiced Kaimai-style Qigong as part of their exercise routine. | Sample Size:  Pre-intervention: N = 55 (I: N = 27; C: N = 28)  Post-intervention assessment: N = 50 (I: N = 27; C: N =23);  Mean Age ± SD:  (N = 55) Total: >60 year-old;  I: 64.54 ± 4.1 year-old;  C: 64.51 ± 3.1 year-old. | Use Kinect-based Kaimai-style Qigong program. Participants exercised three times per week, 30 minutes per session. | Maintain their original lifestyle and did not receive any exercise intervention. | Duration:  12 weeks;  Assessment Timepoints :Baseline and after  the 12-week  Intervention. | FBG; HbA1c; TC; LDL-C; HDL-C; TG; Cognitive Function; Balance; Adverse Events. |
| Höchsmann C et al, 2019 (Switzerland) [20] | 2-arm  Randomized  Trial | Non-insulin-dependent diabetes mellitus;  BMI ≥25 kg/m^2^;  <150 min of moderate-intensity PA per week. | Sample Size:  Pre-intervention: N = 36 (I: N = 18; C: N = 18) ; Post-intervention assessment: N = 35 (I: N = 18; C: N = 17);   Median Age (IQR):  (N = 36) Total: 53-63 year-old;  I: 57 (53, 60);  C: 60 (54, 63) . | A game in a self-developed smartphone includes individualized multidimensional exercise and daily PA (walking). Participants refined game’s storyline through regular in-game PA and the related in-game rewards. | A one-time individual lifestyle counseling(essentially comparable to the content of the game). | Duration:  24 weeks;  Assessment Timepoints : baseline and after the 24-week intervention. | HbA1c; TC; LDL-C; HDL-C; TG; blood pressure (BP) at rest; Total body fat mass; Skeletal muscle mass; aerobic capacity(the maximum oxygen uptake and first ventilatory  threshold );  daily physical activity: steps/d  (through wearing Vivofit 2 accelerometer wristband) |
| Brown SJ et al, 1997 (USA) [21] | 2-arm  Randomized  Trial | 8 -16 years old;  diagnosed insulin-dependent diabetes for ≥ 3 months. | Sample Size:  Post-intervention assessment: N =59 (I: N =31; C: N = 28);  Mean Age ± SD: none. | Play an diabetes education video game *Packy &*  *Marlon.* | Play an entertainment video game with no health content. | Duration: 6 months;  Assessment Timepoints : baseline and after the 3 months, 6 months intervention. | HbA1c;  diabetes knowledge test score (child);  Diabetes self-care rating scales (parent and child);  Urgent visits for  diabetes in past three months. |
| Koohmareh Z et al, 2021 (Iran) [22] | 2-arm  Randomized  Trial | Adult patients; diabetes. | Sample Size:  Pre-intervention: N = 60 (I: N =30; C: N = 30)  Post-intervention assessment: N = 60 (I: N =30; C: N = 30);  Mean Age ± SD:  (N = 60)  I: 44.13 ± 7.91 year-old;  C: 43.93 ± 8.99year-old. | Play the Amoo game 15 minutes per day for 6 weeks.  Intervention group were monitored by a WhatsApp group. | Received educational content similar to the game through a brochure. | Duration: 6 weeks;  Assessment  Timepoints : baseline and after the 6 weeks intervention. | FBG；  test score(based on the game)；  attention to food calorie counts and food glucose level. |
| Kumar VS et al, 2004 (USA) [23] | 2-arm  Randomized  Trial | 8-18 years old;  insulin-treated;  type 1 diabetes or type 2 diabetes. | Sample Size:  Pre-intervention: N =40 (I: N =19; C: N = 21)  Post-intervention assessment: N =40 (I: N =19; C: N = 21);  Mean Age ± SD:(N = 40) ;  I: 13.9± 2.2  year-old;  C: 13.3± 2.8 year-old. | Receiving a handheld personal digital assistant (PDA) fitted with a wireless modem, software for diabetes management, and a game software DiaBetNet. Access the DiaBetNet game daily. | Receiving a handheld personal digital assistant (PDA) fitted with a wireless modem and software for diabetes management. | Duration: 4 weeks.  Assessment Timepoints : baseline, after the 3 months intervention(HbA1c), after the 4 weeks intervention (diabetes knowledge and general satisfaction). | HbA1c;  Diabetes knowledge and general ;  Satisfaction. |
| Rafeezadeh E et al, 2019 (Iran) [24] | 2-arm  Randomized  Trial | 8-12 years old;  type 1 diabetes;  no family history of diabetes. | Sample Size:  Pre-intervention: N = 70 (I: N =35; C: N = 35)  Post-intervention assessment: N = 68 (I: N =34; C: N = 34);  Mean Age ± SD:  (N = 68)  I: 9.9±1.4 year-old;  C: 10.4±1.2 year-old. | Play an educational interactive video game for 12 weeks and at least one hour and a maximum of four hours a week. | - | Duration:  3 months;  Assessment  Timepoints : baseline and after the 3 months intervention. | HbA1c;  scores of self-care;  diet and exercise regimen adherence questionnaire. |
| Batch BC et al, 2021 (USA) [25] | non-RCT | ≥18 years of age;  type 2 diabetes;  HbA1c ≥8% (and <12%) within the past 3 months | Sample Size:  Pre-intervention: N =201 (I: N =100; C: N = 101)  Post-intervention assessment: N = 143(I: N =100; C: N = 43);  Mean Age ± SD:  (N = 201)  I: 56.06±11.00 year-old;  C: 58.52±10.75 year-old. | Downloaded the Time2Focus mobile app (n=100) and interacting with it (regardless of completion of any level).  The app leverages gamification principles to drive patients’ self-management behaviors. | Did not download the app or complete any modules comprised the control group. | Duration: 3 months  Assessment  Timepoints : baseline and after the 3 months intervention. | HbA1c;  Change in Self-Reported. |

Abbreviations: T1DM, type of 1 diabetes mellitus; T2DM, type of 2 diabetes mellitus; RCT, randomized controlled trial; non-RCT, non-randomized controlled trial; HbA1c, glycated hemoglobin A1c; FBG, fasting blood glucose; BMI, body mass index; TC=Total cholesterol; LDL-C, low-density lipoprotein cholesterol; HDL-C, high-density lipoprotein cholesterol; TG, triglycerides; PA; IV, inverse variance; Std., standardized; SMD, standardized mean differences.
